# The accessibility and site selection optimization of elderly care institutions in China: A case study

**DOI:** 10.1093/geroni/igaf122.3434

**Published:** 2025-12-31

**Authors:** Rong Peng, Yuanqing Huang

**Affiliations:** Guangdong University of Finance and Economics, Guangzhou, Guangdong, China (People’s Republic); Guangdong University of Finance and Economics, Guangzhou, Guangdong, China (People’s Republic)

## Abstract

Adjusting and optimizing the spatial layout of elderly care facilities is of great significance to maximize the utilization of elderly care resources for urban residents. Guangzhou is a megacity in southern China and is one of the most economically developed cities in China. The POI data of Guangzhou’s elderly care institutions is used to measure the spatial accessibility of elderly care institutions from the township (street) scale based on the Gaussian based 2-step floating catchment area method (Ga2SFCA). This study constructs the index system for the site selection decision of elderly care institutions, and uses the random forest algorithm to select the grid suitable for the layout of elderly care institutions. The results show that the accessibility of elderly care institutions in Guangzhou’s central area is relatively high, followed by the main urban area and the suburbs. The accuracy of the simulated location results of elderly care institutions and the real location of elderly care institutions is 90.1%, indicating that the random forest model constructed in this study is reliable. From the perspective of the characteristics of the sites suitable for newly built institutions, it is mainly the areas with dense elderly population and the areas with serious shortage of elderly care institutions. The contribution of this study is to construct a site selection decision index system based on POI data, and use the random forest algorithm to optimize site selection decision of elderly care institutions. This study provides a case study in optimizing the layout of elderly care resources.

